# Biochar derived from corn straw affected availability and distribution of soil nutrients and cotton yield

**DOI:** 10.1371/journal.pone.0189924

**Published:** 2018-01-11

**Authors:** Xiaofei Tian, Chengliang Li, Min Zhang, Yongshan Wan, Zhihua Xie, Baocheng Chen, Wenqing Li

**Affiliations:** 1 National Engineering Laboratory for Efficient Utilization of Soil and Fertilizer Resources, National Engineering Technology Research Center for Slow and Controlled Release Fertilizers, College of Resources and Environment, Shandong Agricultural University, Tai’an, Shandong, China; 2 Soil and Water Science Department, Tropical Research & Education Center, University of Florida, Homestead, Florida, United States of America; 3 Jining Academy of Agricultural Sciences, Jining, Shandong, China; RMIT University, AUSTRALIA

## Abstract

Biochar application as a soil amendment has been proposed as a strategy to improve soil fertility and increase crop yields. However, the effects of successive biochar applications on cotton yields and nutrient distribution in soil are not well documented. A three-year field study was conducted to investigate the effects of successive biochar applications at different rates on cotton yield and on the soil nutrient distribution in the 0–100 cm soil profile. Biochar was applied at 0, 5, 10, and 20 t ha^-1^ (expressed as Control, BC5, BC10, and BC20, respectively) for each cotton season, with identical doses of chemical fertilizers. Biochar enhanced the cotton lint yield by 8.0–15.8%, 9.3–13.9%, and 9.2–21.9% in 2013, 2014, and 2015, respectively, and high levels of biochar application achieved high cotton yields each year. Leaching of soil nitrate was reduced, while the pH values, soil organic carbon, total nitrogen (N), and available K content of the 0–20 cm soil layer were increased in 2014 and 2015. However, the changes in the soil available P content were less substantial. This study suggests that successive biochar amendments have the potential to enhance cotton productivity and soil fertility while reducing nitrate leaching.

## Introduction

The application of chemical fertilizers is essential for modern agriculture, contributing approximately 30–50% to increases in crop yields [1]. However, the dependence on soil nutrients in the form of chemical fertilizers and low supplemental organic input into the land has become a major concern in intensive agriculture due to the associated low efficiency of fertilizer utilization and potential environmental pollution [2–3]. These problems are especially pronounced on the North China Plain, where only 45% of the applied nitrogen (N) is absorbed by crops [4–5]. Most of the N applied in agricultural fields is lost, mainly through surface runoff, ammonia volatilization, and NO_3_^−^−N leaching [6–7], resulting in severe soil degradation and groundwater pollution; therefore, effective and comprehensive soil management strategies must be urgently developed not only to improve crop yield and quality but also to enhance soil fertility.

Biochar is a carbon-rich and porous material that is resistant to decomposition in the natural environment due to its condensed structure [8]. Because of its stable organic carbon content, large specific surface area, and negative surface charge [9], biochar has been widely recognized as a beneficial soil amendment for its role in improving soil physical [10], chemical [11], and biological [12–13] properties, as well as in enhancing crop productivity [14–15]. Our review of over 50 reports of research on this subject (Table 1) revealed several interesting points. First, the beneficial role of biochar applications for soil fertility improvements varied with the type of nutrients, the experimental conditions, and the length of the study. For example, although Gaskin et al. [30] found that biochar application directly increased the soil carbon content by adding organic materials (C and N) to the soil, they did not observe any change in the soil P content after two years of field study. Although previous studies showed lower nitrate levels with biochar application, most of them were conducted with column or incubation experiments [24–25]. Comparable field studies were not conclusive on this topic. Major et al. [31] found that the amendment of a low-fertility soil with wood biochar at 20 t ha^-1^ increased the concentration of NO_3_^−^-N in the soil solution. In addition, improved soil fertility or elevated nutrient availability was observed mostly in surface soils [32], with most studies focusing on topsoil at the 0–20 cm depth (Table 1). Very limited research has examined the migration of nutrients or soil carbon into deeper layers in the soil profile with consecutive biochar applications.

**Table 1:** Influence of biochar application on crop yields and soil properties based on the literatures.

Soil type	Crop	Location	Years of experiment	Type of experiment	Biochar type	Biochar rates	Soil depths examined	Major finding	Reference
Wahiawa and Khorat soil	Corn	Thailand	1	Pot	Wood	0–4% w/w	--	- (biomass, first season) + (biomass, second season)	[14]
Sandy soil and loamy soil	Oats	Germany	1	Pot	Wood	0–50 wt.%	--	+ (grain yield)	[15]
Calcareous inceptisol	Maize	China	2	Field	Straw	0–40 tha^-1^	0–15 cm	+11.9%-35.4%(maize yield)	[16]
Light clay	Maize	Australia	1	Field	Wood	0–25 tha^-1^	0–12 cm	+8%-29% (grain yield)	[7]
Haplic Luvisol	Wheat	Spain	1	Pot	Straw and olive-tree	0–2.5% w/w	--	+10%-100%(root biomass)	[17]
Clay loam	Rice	China	2	Field	Pine	0–16 gkg^-1^	0–20 cm	+11.3%-21.6%(rice yield)	[18]
Light clay	Maize	Australia	1	Field	Wood	0–25 tha^-1^	0–12 cm	+13%-29%(grain yield)	[13]
Entic Halpudept	Rice	China	2	Field	Straw	0–40 tha^-1^	0–15 cm	+ 9%-28% (grain yield)	[19]
Clay loam	Maize	Colombia	4	Field	Wood	0–20 tha^-1^	0–30 cm	+0%-140% (grain yield)	[20]
Sandy loam	Grass	China	3	Field	Pine	0–16 gkg^-1^	0–40 cm	+2.7 g/kg-10.7 g/kg (soil organic carbon)	[18]
-	Corn or sorghum	USA	3	Field	Wood	0-20Mgha^-1^	0–30 cm	+41%-46% (soil organic carbon)	[21]
Calcareous inceptisol	Maize	China	2	Field	Straw	0–40 tha^-1^	0–15 cm	+4.9 g/kg-12.8 g/kg (soil organic carbon)	[16]
Silt loam	Wheat and maize	China	3	Field	Mushroom residue	0–90 tha^-1^	0–20	+44%-215%(soil organic carbon)	[22]
Clay loam	Cotton	China	1	Field	Straw	0–4.5 tha^-1^	0–20	+8%-109%(soil organic carbon)	[23]
Calcareous inceptisol	Maize	China	2	Field	Straw	0–40 tha^-1^	0–15 cm	+4%-12%(total N)	[16]
Sandy loam	Grass	China	3	Field	Wood	0-20Mgha^-1^	0–40 cm	+0.2 g/kg-0.8 g/kg (total N)	[21]
Loam soil	-	India	2	Column	Corn	0–20 gkg^-1^	0–40 cm	-14%-32%(nitrate loss)	[24]
Sandy soil	-	USA	1	Incubation	Wood and sraw	-	--	-34%(nitrate loss)	[25]
Loam soil	Cotton	China	2	Field	Straw	0–12 tha^-1^	--	-21%(NH_3_ volatilization)	[26]
-	Oat	Finland	5	Field	-	9 tha^-1^	0–20	+96% (CH_4_ uptake)	[27]
Haplic Luvisol	sunflower	Spain	1	Pot	Wood and sraw	0–7.5% w/w	--	+ 0.5-1units (pH)	[28]
Sandy clay	Maize	UK	3	Field	Wood	0–50 tha^-1^	0–20 cm	+0.32 units (pH)	[29]
Sand	Wheat	Australia	1	Pot	Wood	0–25 tha^-1^	--	- (microbial biomass C:N ratio)	[13]
Silt loam	Wheat and maize	China	3	Field	Mushroom residue	0–90 tha^-1^	0–20	+38%-84%(C/N)	[22]
Light clay	Maize	Australia	1	Field	Wood	0–25 tha^-1^	0–12 cm	+9%-25% (soil water contents)	[7]
loamy sand	Corn	USA	2	Field	PPeanut hull	0-22Mgha^-1^	0–30 cm	Not affected (Soil P)	[30]
loamy sand	Corn	USA	2	Field	Peanut hull	0-22Mgha^-1^	0–30 cm	+17%-98% (Soil K)	[30]
Clay loam	Maize	Colombia	4	Field	Wood	0–20 tha^-1^	0–30 cm	+77%-320%(available Ca and Mg)	[20]
Clay loam	Cotton	China	1	Field	Straw	0–4.5 tha^-1^	0–20	+19%-27%(N use efficiency)	[23]

Note: -- indicates no data.

+ increase.

- decrease.

Second, increases in crop yields with biochar application have been studied mostly with a single biochar application [33–34]. For example, Liao et al. [23] verified that a single amendment of 4.5 t ha^-1^ biochar significantly increased cotton yields, by 24–37%, in a one-year field study; in contrast, Major et al. [20] noted that wood biochar application at a single dose of 20 ha^-1^ had no obvious influence on maize yields in the first year, although crop yields in the subsequent three years were significantly increased. Zhang et al. [19] affirmed that biochar application enhanced rice yields by 10% in the first cycle and by 9.5–29.0% in the subsequent cycle. Little information is available about the effects of successive biochar applications on crop yields through long-term field observations.

Overall, the effects of biochar applications for improving soil fertility and increasing crop productivity are complex and depend on the soil type, biochar properties, biochar application rate, chemical fertilizers used, and the years examined [19–20, 35]. Our study contributes to the existing literature through a comprehensive examination of the soil nutrient distribution along 1 m depth of the soil profile and the enhancement of crop yield and quality with three successive years of biochar applications in the field. This general assessment depends on details of the interactions between biochar, soil, and application times. We hypothesized that successive applications of biochar will have significant incremental benefits in (i) cotton yield and quality, (ii) overall soil fertility, and (iii) decreased NO_3_^−^−N leaching. Our three-year field experiment was conducted on the North China Plain. Specifically, the objectives of this study were (1) to investigate the long-term effects of different rates of biochar application on cotton yield, fiber quality, and topsoil fertility, with identical doses of controlled release urea; and (2) to study the dynamic changes in response to biochar application in the leaching of soil NO_3_^−^−N and NH_4_^+^-N and the distribution of soil pH, organic carbon, total N, available K, and available P across the 0–100 cm soil profile.

## Materials and methods

### Experimental site and plant cultivation

The study was performed at Zhoulianchi Village (34°58′N, 116°10′E), Jinxiang County, Shandong Province, China, from May 2013 to October 2015 under a cotton-garlic intercropping system. As a local conventional cropping system, cultivation of cotton in the summer and garlic in the winter has been popular at this site since the 1980s. This region has a Dwa climate according to Köppen climatic classification, with low temperatures below 0°C in the winter and high temperatures above 40°C in the summer. Annual precipitation is 700 mm, most of which falls from June to August. The predominant soil of the experimental site is classified as an Inceptisol according to U.S.D.A. Soil Taxonomy [36]. The basic properties of the experimental soil at the initiation of the study are described in Table 2.

**Table 2:** Basic properties of 0–20 and 20–40 cm soil before planting in 2012.

Depth (cm)	pH (water)	Organic C (g kg^-1^)	Total N (g kg^-1^)	NH_4_^+^-N (mg kg^-1^)	NO_3_^−^−N (mg kg^-1^)	Available P (mg kg^-1^)	Available K (mg kg^-1^)
0–20	7.67	8.74	0.61	10.68	18.09	34.70	97.5
20–40	7.70	6.59	0.39	10.34	17.54	14.32	101.2

Note: The pH of the soil was determined at a soil/water ratio of 1:2.5.

The feedstock for the biochar used in this study was corn straw, which was collected from an experimental site at the National Engineering Laboratory for Efficient Utilization of Soil and Fertilizer Resources in Tai'an, Shandong province (34°20′N, 117°13′E). The biochar was produced from the slow pyrolysis of corn straw at 450°C, with a residence time of 2 h under oxygen-limited conditions in a programmed pyrolysis furnace (Taian Hongtai, Inc., Shandong, China), followed by cooling at room temperature for 24 h. The basic properties of the biochar are pH (H_2_O) 10.3; ash content 109.1 g kg^-1^; CEC 40.1 cmol kg^-1^; and total C, N, P, K, Ca, and Mg contents of 890.5, 7.2, 4.3, 15.2, 3.20, and 1.13 g kg^-1^, respectively.

The cotton cultivar was ‘Lu Yanmian 28’, which has been widely adopted in northern and central China. The chemical fertilizers, including polymer coating of sulfur-coated urea (PSCU, 35% N), polymer-coated urea (PCU, 43% N), urea (N 46%), diammonium phosphate (DAP, 48% P_2_O_5_, and 16% N), and potassium sulfate (KPS, 50% K_2_O), were provided by Kingenta Ecological Engineering Group Co., Ltd., China.

### Experimental design and field management

The experiment was conducted as a randomized complete block design with three replications. The four treatments were (1) chemical fertilizer without biochar application (Control); (2) chemical fertilizer with biochar applied at 5t ha^-1^ each cotton season (BC5); (3) chemical fertilizer with biochar applied at 10 t ha^-1^ each cotton season (BC10); and (4) chemical fertilizer with biochar applied at 20 t ha^-1^ each cotton season (BC20). The chemical fertilizers used for cotton and garlic were the same. In all treatments, 220 kg ha^-1^ N, 180 kg ha^-1^ P_2_O_5_, and 90 kg ha^-1^ K_2_O were basally applied during each growing season. Of the total N, 50% was applied as PCU and PSCU in equal proportion, and the remainder was supplied as urea and DAP. DAP was also used as the P fertilizer and KPS as the K fertilizer.

Under the cotton-garlic intercropping system, cotton seedlings were transplanted into the garlic row spaces before the garlic harvest. Thus, the two crops were growing together side by side in the field for approximately 30–40 days. Approximately 20–25 days after the garlic harvest, chemical fertilizers and biochar were applied via deep-strip tilling in the soil at a depth of 15 cm. No irrigation or chemical fertilizer top-dressing was used during the entire cotton growth period.

Three replicated trial plots (4.4 m×5 m) were established and separated by buffer rows that were 0.7 m wide, each with an irrigation and drainage outlet. Cotton was seeded in April and transplanted in the field in mid-May, with a row space of 110 cm, a plant space of 35 cm, and an actual density of 20,800 plants ha^−1^(S1 Fig). After the cotton was uprooted in early October, before all the bolls opened, chemical fertilizers for the garlic were spread on the surface and plowed to a depth of over 15 cm before sowing.

### Sampling and measurement

At maturity, the seed yield was measured by the arithmetic product of boll weight, the average number of bolls, and plant density. Before the cotton was uprooted, 100 open bolls (>2 cm in diameter) were randomly picked by hand from each plot and air dried before the boll weight was measured, after which the lint percentage and fiber quality were measured. Meanwhile, 20 consecutive plants in the middle two rows were used to survey boll numbers. Then, the fiber samples were sent to the cotton quality supervision and inspection center (Henan) to determine the fiber quality parameters (e.g., micronaire, fiber length, fiber strength, length uniformity index, and fiber elongation) of each sample.

When the cotton was uprooted each year, soil samples were taken from each plot at 20 cm intervals from soil depths of 0 to 100 cm. Fresh soil samples of the same depth from five random locations per plot were mixed as a composite sample. Part of each sample was stored fresh in a 4°C refrigerator, and the remainder was air-dried and sieved through 2.0-mm and 0.25-mm sieves.

The contents of NO_3_^−^−N and NH_4_^+^-N (extracted with 0.01 M CaCl_2_) in fresh soil samples were analyzed using an AA3-A001-02E auto-analyzer (Bran-Luebbe, Germany) within 48 hours after collection. The organic carbon content was measured using a WR112 Leco carbon detector (LECO Corp., Michigan, USA). The total N content was measured using the Kjeldahl digestion method [37]. Soil pH was measured at a 1:2.5 (w/v) ratio of soil to CO_2_-free distilled water using a pH meter (PB-10, Sartorius AG, Germany). The soil available P content was determined using the Olsen-P method based on the extraction of air-dry soil with 0.5 M NaHCO_3_ at pH 8.5 and the spectrophotometric method. The soil available K content was measured using the CH_3_COONH_4_ extraction method with a flame photometer.

### Statistical analysis

Two-way analysis of variation (ANOVA) was performed to determine the effects of the biochar treatment, year (application times), and their interaction on yield, fiber quality, and soil properties. One-way ANOVA was performed to assess the significant differences of cotton growth, quality, and soil nutrients between different treatments within the same year. ANOVAs and mean separation tests (Duncan’s multiple range test at the 5% probability level) were performed using the Statistical Analysis System package, version 9.2 (2010, SAS Institute Cary, NC). Means and standard errors were calculated to assemble graphs using Sigma Plot software, version 10 (MMIV Systat Software, Inc., San Jose, CA).

## Results

### Effects on cotton growth and yield

Analysis of variance showed that the biochar application and years had significant (*p* < 0.01) effects on cotton yields though the interactions between them were not significant (Table 3). The cotton yield increased with the increasing rate of biochar amendments and with increasing application times within the same rate. In 2013, the seed yield of BC10 and BC20 treatments increased by 10.3% and 17.1%, respectively, over that of the control (*p* < 0.05), although the yield increase with BC5 was not significant. The seed yield of the BC20 treatment increased by 6.2% and 8.3%, respectively, over BC10 and BC5 (*p* < 0.05), but no significant difference was detected between BC5 and BC10. In 2014, the seed yield increased over the control by 9.6%, 12.2%, and 13.5%, respectively, for the BC5, BC10, and BC20 treatments. In 2015, the corresponding increase was 8.1%, 15.4%, and 18.6%, respectively, for the three doses of biochar application. Similar trends were observed with cotton lint yield among all treatments (Table 3).

**Table 3:** Cotton yield and yield components in response to biochar application at the rate of 0, 5, 10 or 20 t ha^−1^ in 2013, 2014and 2015.

Year	Treatments	Height (cm)	Stem diameter (cm)	Branches	Bolls (per ha^-1^)	Boll weight (g boll^-1^)	Lint percentage (%)	Seed yields (kg ha^-1^)	Lint yields (kg ha^-1^)	Lint yields vs. Control (%)
2013	Control	108.7 a	1.82 a	16.5 a	824852 b	6.19 b	39.9 a	5046.2 c	2016.8 c	-
	BC5	109.3 a	1.83 a	16.3 a	882875 b	6.18 b	39.9 a	5453.5 bc	2177.9 b	7.98
	BC10	105.2 a	1.87 a	16.8 a	879790 b	6.32 ab	39.6 a	5564.0 b	2201.5 ab	9.18
	BC20	116.0 a	1.87 a	16.5 a	924640 a	6.39 a	38.9 a	5906.5 a	2334.9 a	15.77
2014	Control	119.2 b	1.78 a	16.5 a	864614 b	6.48 c	44.0 b	5602.7 b	2529.7 b	-
	BC5	126.2 a	1.74 a	16.3 a	930090a	6.50 c	44.8 ab	6138.6 a	2763.7 a	9.25
	BC10	126.2 a	1.72 a	16.2 a	928242 a	6.77 b	45.1 ab	6284.2 a	2834.7 a	12.06
	BC20	123.9 a	1.72 a	16.8 a	900439 a	7.06 a	45.4 a	6357.1 a	2880.1 a	13.87
2015	Control	116.3 b	1.72 a	15.3 a	842682 b	6.77 a	41.8 a	5704.9 c	2386.58 c	-
	BC5	124.9 a	1.77 a	15.3 a	903826 a	6.82 a	42.3 a	6164.1 b	2606.87 b	9.22
	BC10	121.5 a	1.84 a	16.0 a	917048 a	7.18 a	42.8 a	6584.4 a	2819.14 a	18.14
	BC20	124.9 a	1.88 a	16.0 a	921841 a	7.34 a	43.0 a	6766.3 a	2909.93 a	21.92
Source of variance										
Year		0.0001	0.0021	0.0007	0.0147	0.0001	0.0001	0.0001	0.0001	-
Treatment		0.0371	0.3259	0.8868	0.0001	0.0001	0.5783	0.0001	0.0001	-
Year×Treatment		0.2972	0.1655	0.0274	0.0984	0.4379	0.3265	0.1378	0.0906	-

Note: Control, BC5, BC10 and BC20 indicate biochar amendment rate of 0, 5, 10 and 20 t ha^-1^.

Means followed by the same letters in the columns are not significantly different according to Duncan’s test at 5% significance level in the same year.

“-”indicates no data.

Plant height was considerably affected by biochar applications and years butwith no significant interaction between those factors (Table 3). The stem diameter and number of branches were not markedly affected by the biochar application rate, with the stem diameter maintained at 1.7–1.9 cm and branches of 16–18 per plant^-1^. In 2013, the BC20 treatment possessed the highest boll number, 7.6–8.8% more than other treatments. In 2014, the highest boll number appeared in the BC10 and BC20 treatments, and no pronounced difference in boll number was observed between BC5 and the control. In 2014, the highest boll weight occurred in BC20, followed by BC10, and the lint percentage was significantly higher in the BC20 treatment than in the control.

### Effects on cotton fiber quality

Analysis of variance showed that the biochar amendments and years had significant (*p*< 0.01) effects on cotton fiber length and fiber strength (Table 4). Fiber length was significantly greater in the BC20 treatment than in the control in both 2014 and 2015. However, no significant difference in fiber length was observed among all treatments in 2013. The fiber micronaire was not affected by biochar application in 2013 or 2014, whereas it decreased with biochar applications in 2015. Fiber uniformity was significantly greater in 2015 than in 2013 and 2014. Fiber strength was significantly greater in the BC20 treatment than in the control in 2013 and 2014, and it was significantly improved with biochar application in 2015. Fiber elongation greatly increased in the BC10 and BC20 treatments compared with the control in 2014.

**Table 4:** Fiber quality in response to biochar application at different rates.

Year	Treatments	Length (mm)	Uniformity (%)	Strength (cNtex^-1^)	Micronaire value	Elongation rate (%)
2013	Control	30.38 a	86.07 a	28.32b	5.03 a	5.50 a
	BC5	30.24 a	86.13 a	28.62 ab	5.00 a	5.67 a
	BC10	30.38 a	86.13 a	28.71 ab	4.74 a	5.65 a
	BC20	30.31 a	86.37 a	30.25 a	4.85 a	5.70 a
2014	Control	28.13 b	84.37 a	27.40 b	4.70 a	6.97 b
	BC5	28.13 b	85.03 a	29.37 ab	4.67 a	7.17 ab
	BC10	28.53 ab	85.13 a	29.33 ab	4.63 a	7.67 a
	BC20	30.10 a	85.83 a	29.83 a	4.47 a	7.67 a
2015	Control	29.03 b	85.10 a	27.93 b	4.87 a	7.03 b
	BC5	30.80 a	85.60 a	28.23 ab	4.87 a	7.17 a
	BC10	30.63 a	85.40 a	28.53 a	4.83 a	7.23 a
	BC20	30.87 a	85.50 a	28.50 a	4.67 b	7.53 a
Source of variance						
Year		0.0001	0.0303	0.4601	0.0002	0.0001
Treatment		0.0267	0.4762	0.5112	0.2401	0.2565
Year×Treatment		0.2219	0.9475	0.6186	0.3292	0.9594

Note: Control, BC5, BC10 and BC20 indicate biochar amendment rate of 0, 5, 10 and 20 t ha^-1^.

Means followed by the same letters in the columns are not significantly different according to Duncan’s test at 5% significance level in the same year.

### Soil organic carbon and total N content

**Fig 1:**
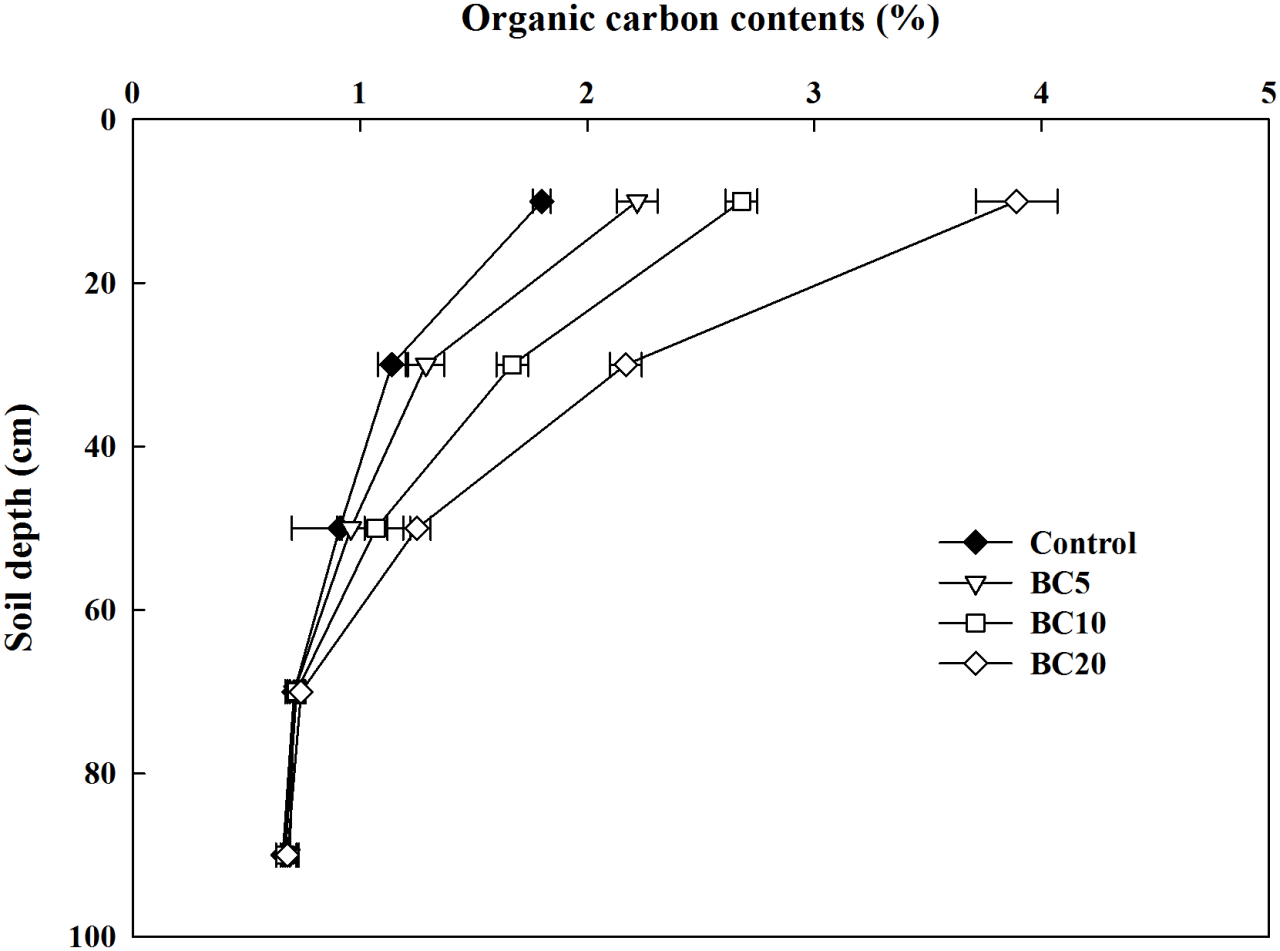
Soil total organic carbon in 0–100 cm soil after cotton harvested in 2015.

After three years of fertilization, the total organic carbon content in the soil decreased with increasing depth for all treatments (Fig 1). In the 0–40 cm soil layer, the total organic carbon increased with the biochar application rate after three years of biochar applications, following the sequence of BC20 > BC10 > BC5 > control (*p* < 0.05). Generally, the soil organic carbon in the 0–20 cm soil layer increased the most. Furthermore, the soil organic carbon content in the 40–60 cm soil layer was significantly higher in the BC20 treatment than in the control.

**Fig 2:**
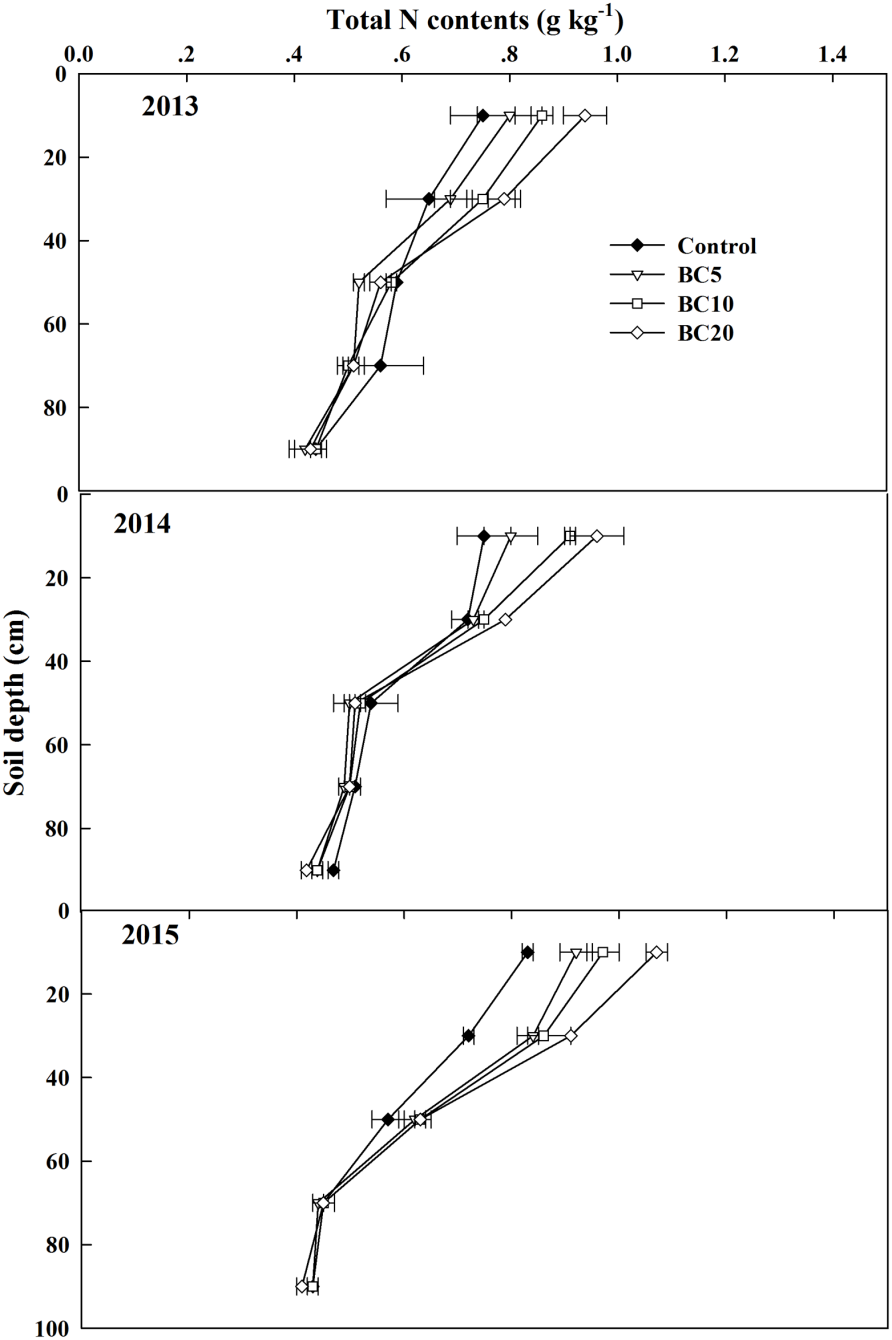
Soil total Nin 0–100 cm soil after cotton harvested in 2013, 2014and 2015.

The total N content was considerably affected by biochar treatments and years, as well as by their interaction, except in the 60–100 cm soil layer (S1 Table). As with the soil organic carbon content trend, the total N content decreased with increasing depth in the 0–100 cm profile (Fig 2). In 2015, the 0–40 cm soil layer showed significantly higher total soil N contents in the biochar application treatments than in the control. No obvious difference between the control and BC5 treatments was observed in 2014. In all three years, total soil N at 20–40 cm was significantly greater in the BC20 treatment than in the control. Furthermore, at soil depths below 40 cm in the profile, no significant differences were seen among all treatments.

### Soil inorganic N content

**Fig 3:**
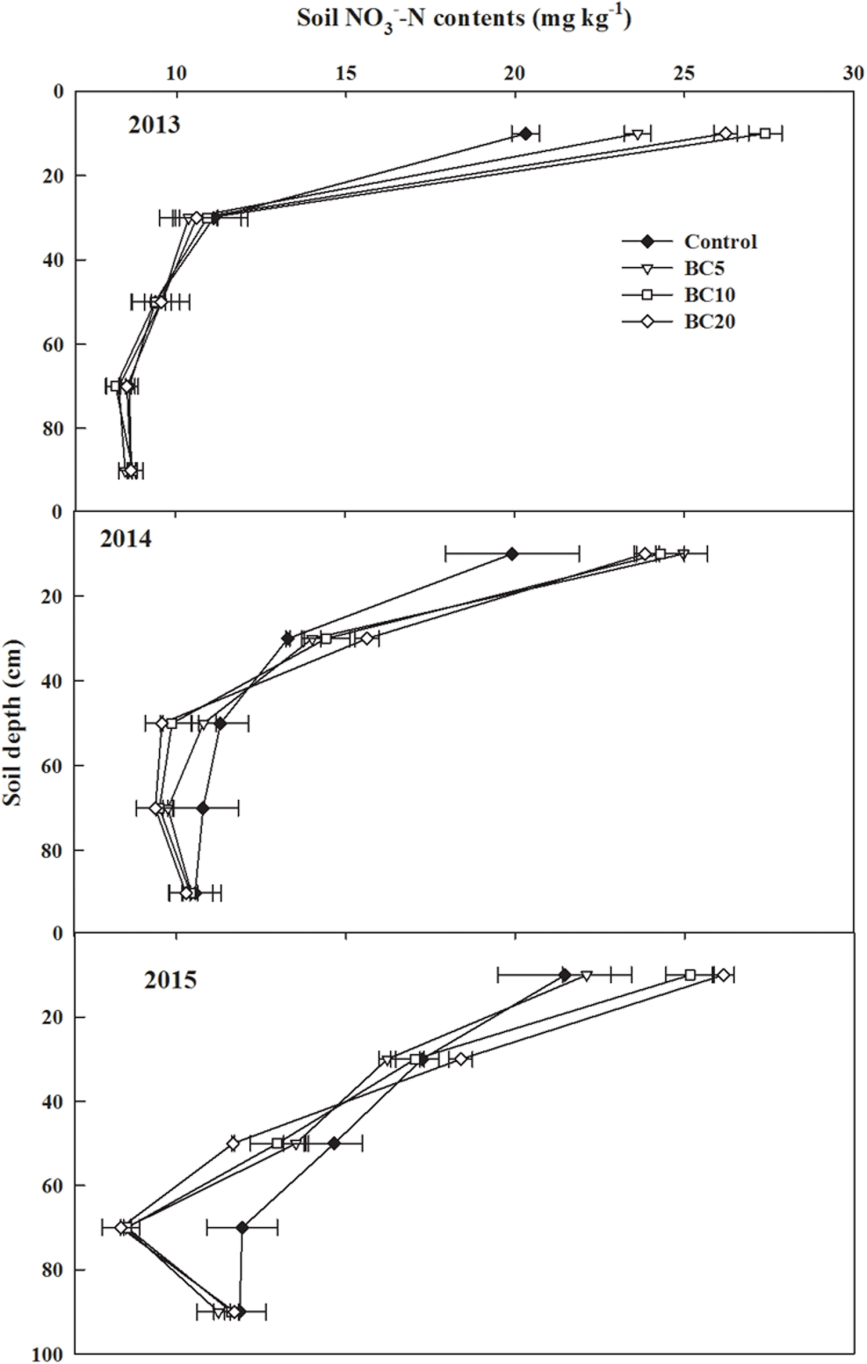
Soil NO_3_^−^−Nin 0–100 cm soil after cotton harvested in 2013, 2014 and 2015.

Biochar applications, years, and their interactions significantly affected the soil NO_3_^−^−N content (S1 Table). The NO_3_^−^−N content decreased with depth in the 0–100 cm soil profile for all treatments. Compared with the control, the 0–20 cm soil profile showed significantly greater NO_3_^−^−N content with biochar application. At other depths, no significant difference in the NO_3_^−^−N content was observed among the treatments in 2013 (Fig 3). Furthermore, the NO_3_^−^−N content of the BC20 treatment in the 0–40 cm soil layer was significantly higher than in the control, but the opposite trend was observed in the 40–60 cm soil layer in 2014 and in the 60–80 cm soil layer in 2015, indicating that successive applications of biochar amendments maintained higher levels of mineral N in the topsoil to feed plants. In addition, biochar amendments decreased the nitrate N content in deeper soil under long-term fertilization.

**Fig 4:**
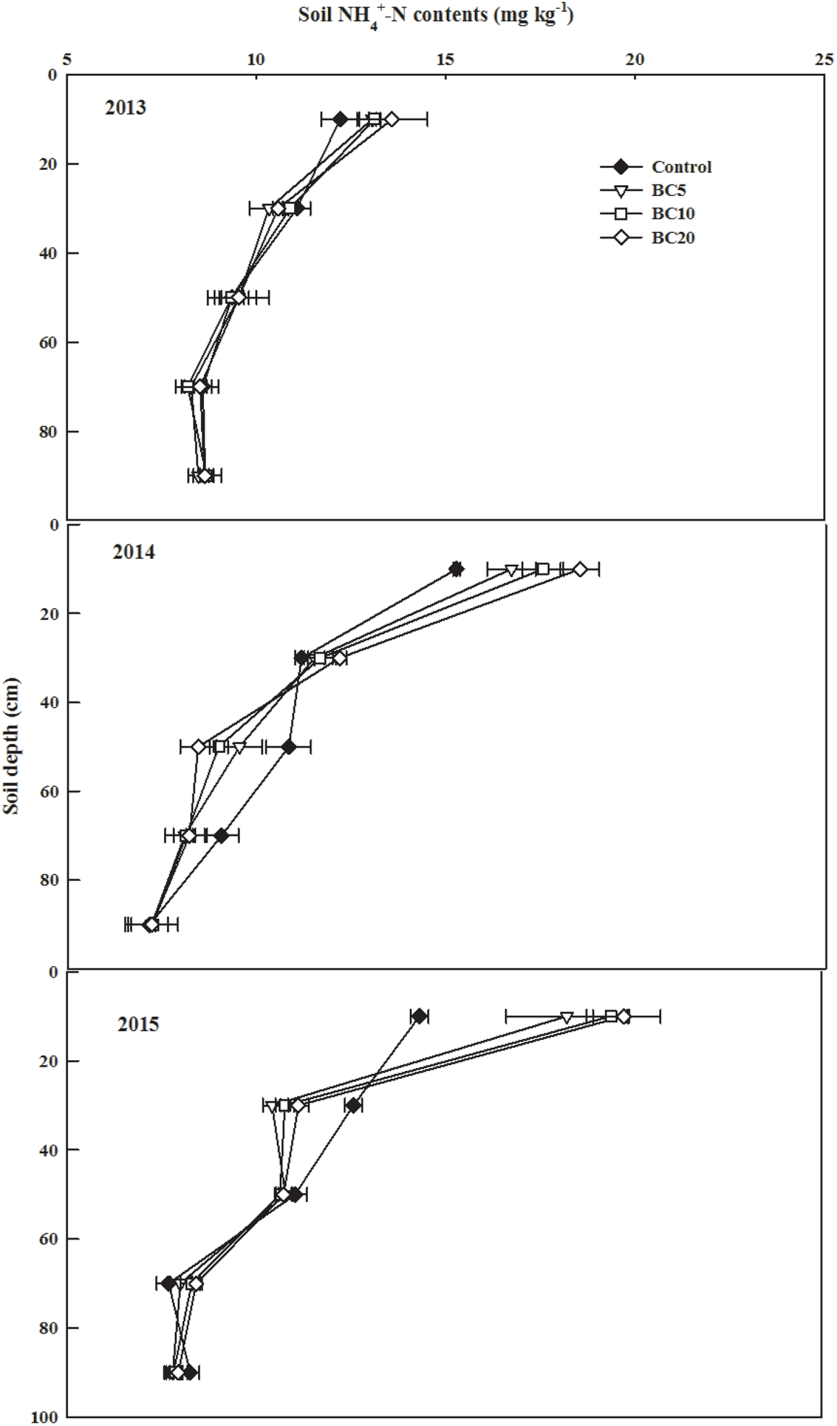
Soil NH_4_^+^−Nin 0–100 cm soil after cotton harvested in 2013, 2014and 2015.

Significant effects on the soil profile NH_4_^+^-N content were observed for biochar applications, years, and their interaction (S1 Table). The NH_4_^+^-N content decreased with the depth of soil for all treatments in all three years (Fig 4). The NH_4_^+^-N contents in the biochar application treatments were significantly greater than in the control in the 0–40 cm soil layer in 2014, but no obvious differences among all treatments were found in 2015. Furthermore, the NH_4_^+^-N contents of the BC5, BC10, and BC20 treatments were significantly higher in the 20–40 cm soil layer than in the control in 2015. Meanwhile, the NH_4_^+^-N content in the 60–100 cm soil layer was not significantly affected by biochar applications in comparison with the control in 2014 and 2015.

### Soil pH values, available P, and available K content

Analysis of variance showed that the addition of biochar, years, and years by treatment interactions had significant (*p* < 0.01) effects on the soil pH in the 0–20 and 20–40 cm soil layers (Table 5). The pH value increased with soil depth in the 0–100 cm soil layer for all treatments in all three years. Compared with the control, the pH values of the biochar application treatments significantly increased in 2014 and 2015. Furthermore, the pH in the topsoil (0–20 cm) was higher in the BC20 treatment than in the BC5 and BC10 treatments in 2014 and 2015. Moreover, the biochar applications significantly increased the pH values of the 20–40 cm soil layer in 2015.

**Table 5:** Soil pH values after cotton harvested in 2013, 2014 and 2015.

Year	Treatments	Soil depths (cm)				
		0–20	20–40	40–60	60–80	80–100
2013	Control	7.74 b	7.89 a	8.00 a	8.10 a	8.18 a
	BC5	7.76 ab	7.88 a	8.00 a	8.10 a	8.19 a
	BC10	7.79 ab	7.90 a	8.05 a	8.10 a	8.18 a
	BC20	7.83 a	7.90 a	8.04 a	8.12 a	8.20 a
2014	Control	7.72 c	7.95 a	7.88 a	8.13 a	8.19 a
	BC5	7.81 b	7.98 a	7.93 a	8.13 a	8.2 a
	BC10	7.81 b	7.95 a	7.96 a	8.14 a	8.21 a
	BC20	7.88 a	7.97 a	7.96 a	8.16 a	8.2 a
2015	Control	7.58 c	7.73 b	7.96 a	8.12 a	8.16 a
	BC5	7.83 b	7.99 a	8.00 a	8.14 a	8.17 a
	BC10	7.86 b	8.00 a	7.98 a	8.12 a	8.17 a
	BC20	7.90 a	8.01 a	7.99 a	8.13 a	8.17 a
Source of variance						
Year		0.2042	0.0001	0.0421	0.0036	0.0008
Treatment		0.0001	0.0001	0.0699	0.2731	0.0916
Year×Treatment		0.0001	0.0002	0.7533	0.8072	0.8010

Note: Control, BC5, BC10 and BC20 indicate biochar amendment rate of 0, 5, 10 and 20 t ha^-1^.

Means followed by the same letters in the columns are not significantly different according to Duncan’s test at 5% significance level in the same year.

Analysis of variance showed that the addition of biochar, years, and years by treatment interactions had significant (*p* < 0.05) effects on the soil available K and available P contents in the 0–20 and 20–40 cm soil layers (Table 6). In general, the soil available P and available K contents in the 0–20 cm soil layer decreased with time and with the depth of soil in the 0–100 cm soil depths (Table 7). In 2014, the soil available P content of the BC20 and BC10 treatments was significantly lower than in BC5 and the control. In contrast, the BC10 and BC20 treatments significantly increased the soil available K content in the 0–20 cm soil layer compared with the control in all three years. In addition, the available K content of the 20–40 cm soil layer significantly increased with the BC10 and BC20 treatments in 2014.

**Table 6:** Soil available P contents after cotton harvested in 2013, 2014 and 2015 (mg kg^-1^).

Year	Treatments	Soil depths (cm)				
		0–20	20–40	40–60	60–80	80–100
2013	Control	32.07 a	19.92 a	9.54 a	5.48 a	8.62 a
	BC5	31.61 a	17.99 a	8.70 a	5.37 a	8.53 a
	BC10	30.97 a	17.29 a	8.44 a	5.42 a	8.75 a
	BC20	29.98 a	15.76 a	7.85 a	5.36 a	8.53 a
2014	Control	31.24 a	17.24 a	8.65 a	4.10 a	7.69 a
	BC5	30.19 a	16.35 a	8.65 a	4.69 a	7.86 a
	BC10	27.58 b	15.48 a	8.61 a	4.10 a	7.79 a
	BC20	26.11 b	14.46 a	8.31 a	4.84 a	7.67 a
2015	Control	27.92 a	14.24 a	7.65 a	7.34 a	7.64 a
	BC5	27.89 a	14.25 a	7.53 a	7.35 a	7.66 a
	BC10	26.58 ab	13.71 a	7.62 a	7.37 a	7.59 a
	BC20	25.43 b	13.35 a	7.52 a	7.40 a	7.67 a
Source of variance						
Year		0.0005	0.0001	0.0066	0.0001	0.0026
Treatment		0.0204	0.3069	0.3281	0.8024	0.9935
Year×Treatment		0.8770	0.8505	0.7223	0.8386	0.9991

Note: Control, BC5, BC10 and BC20 indicate biochar amendment rate of 0, 5, 10 and 20 t ha^-1^.

Means followed by the same letters in the columns are not significantly different according to Duncan’s test at 5% significance level in the same year.

**Table 7:** Soil available K contents after cotton harvested in 2013, 2014 and 2015 (mg kg^-1^).

Year	Treatments	Soil depths (cm)				
		0–20	20–40	40–60	60–80	80–100
2013	Control	117.89 c	104.74 b	90.28 a	96.13 a	82.42 a
	BC5	127.32 bc	115.71 a	89.70 a	89.78 a	80.40 a
	BC10	138.52 ab	118.36 a	91.96 a	91.32 a	80.93 a
	BC20	146.46 a	124.41 a	92.64 a	91.01 a	91.19 a
2014	Control	116.06 c	109.53 a	90.34 a	90.77 a	87.78 a
	BC5	124.68 bc	111.08 a	95.82 a	87.24 a	86.69 a
	BC10	133.60 ab	113.88 a	98.81 a	86.56 a	87.38 a
	BC20	142.65 a	117.02 a	95.46 a	84.88 a	87.65 a
2015	Control	116.99 b	100.54 c	87.42 a	90.65 a	89.61 a
	BC5	128.14 ab	108.99 bc	92.36 a	90.24 a	88.93 a
	BC10	132.83 a	112.13 ab	95.35 a	89.56 a	89.62 a
	BC20	139.67 a	118.26 a	95.33 a	87.88 a	89.89 a
Source of variance						
Year		0.6603	0.0332	0.4502	0.5969	0.0200
Treatment		0.0001	0.0001	0.3707	0.9646	0.9836
Year×Treatment		0.9935	0.4837	0.9797	0.8041	0.9999

Note: Control, BC5, BC10 and BC20 indicate biochar amendment rate of 0, 5, 10 and 20 t ha^-1^.

Means followed by the same letters in the columns are not significantly different according to Duncan’s test at 5% significance level in the same year.

## Discussion

### Effects of successive biochar application on cotton yield and fiber quality

In this study, biochar applications to silt loam soil increased seed cotton yields by 8.1–17.1%, 9.6–13.5%, and 8.1–18.6% in 2013, 2014, and 2015, respectively. Similarly, in a previous study, the maize yield did not increase with 20 t ha^-1^ biochar amendment in the first year, but it increased by 28–140% in the following three years [20]. The increases in cotton yield could be attributed to the addition of nutrients along with biochar, as well as to associated improvement in soil structure and moisture conditions [27, 38]. Meanwhile, many experiments have also reported improvements in the soil water-holding capacity after biochar amendment [39]. Thus, the improvement in soil moisture conditions may be another contributor to increases in cotton yield in water-limited cropland.

Based on a meta-analysis of literature data, Liu et al. [40] demonstrated a convincing positive response of crop yields to biochar application, with a few negative responses limited to specific circumstances. In a pot experiment, Butnan et al. [14] proved that a single amendment of 2% w/w biochar decreased corn biomass accumulation in the first cycle and increased biomass accumulation in the second season. Rajkovich et al. [39] also reported that a single dose of food waste biochar at 90 t ha^-1^ resulted in an 80% decline in crop productivity. In this study, the total amendments of 15, 30, and 60 t ha^-1^ biochar in three treatments (2013, 2014, and 2015), rather than a single application, resulted in a steady increase in the cotton lint yield. Hence, successive applications of biochar may be a better approach under field conditions. However, further studies should be conducted to verify the differences between a single biochar application and separate biochar applications and the total amounts of cotton growth under field conditions.

Producing longer and stronger cotton fiber with a suitable micronaire is important for cotton market preference. In this study, fiber uniformity, which may be an intrinsic genetic quality, was not affected by biochar amendments. However, in 2014 and 2015, BC20 showed significantly higher fiber length and strength than the control. Numerous studies have found that fiber length and fiber strength were adversely affected by K [41] and N deficiency [42] but were less sensitive to soil available P [43]. The observed increases in fiber length and strength in this study were probably because biochar applications increased the soil available K content and inorganic N content. Although the effect of the 20 t ha^-1^ biochar application on the micronaire was significant (*p* < 0.01), only in 2015 was the micronaire value in the biochar treatments significantly lower than in the control treatment. Further studies are required to elucidate the mechanisms of how biochar changes the fiber micronaire value of cotton.

### Successive biochar application decreased nitrogen leaching and affected soil properties

In this study, biochar applications increased the NO_3_^−^−N content at the soil depth of 0–20 cm but decreased its content at 60–80 cm soil depth in 2015 (Fig 4), indicating that successive applications of biochar maintained more mineral N in the topsoil to feed plants under long-term fertilization. Yao et al. [25] indicated that amendments with biochar derived from peanut hulls (2% of the soil, w/w) reduced the leaching of NO_3_^−^−N and NH_4_^+^-N by 34% and 14%, respectively, in a soil column experiment. The mechanisms responsible for reduced NO_3_^−^−N leaching through biochar applications may be related to the functional properties of biochar, such as its large surface area, highly porous structure, and strong ion exchange capacity [44]. Biochar application in farmlands increased the residence time of NO_3_^−^−N in arable soil and provided greater opportunity for crops to absorb NO_3_^−^−N [45], which then decreased soil NO_3_^−^−N leaching potential. The soil NH_4_^+^-N content in the 0–20 cm soil layer increased with biochar application in 2014 and 2015, whereas no significant differences were observed in the 40–100 cm soil layer. Similar results were reported by Agegnehu et al. [7], who found that biochar particles adsorbed NH_4_^+^, thus decreasing soil NH_4_^+^-N loss and increased its concentration.

The effects of biochar on soil properties vary widely depending on the characteristics of both the underlying soil and the biochar [32]. In the present study, the organic carbon content of the 0–20 cm soil layer increased with increasing rates of biochar application, which was consistent with previous findings [14, 22]. Dong et al. [22] demonstrated, based on data from a three-year field study with a single biochar application, that biochar derived from mushroom waste enhanced the levels of water-soluble organic C during rice/wheat seasons compared with the control. In this study, 73.9–93.9% of the carbon from biochar addition was detected in the 0–20 cm soil profile after three years of successive applications. Meanwhile, the organic soil carbon content was also significantly higher in the 20–40 cm soil than in the control after three years of amendments. This is consistent with previous field studies and may have been caused by the downward movement of the fine biochar particles into the subsoil by earthworm activity, root growth, and leaching [46].

Biochar can improve the physical and chemical properties of the soil, change the soil pH, and alter soil microbial populations, all of which can affect nitrogen cycling [24–25]. Like soil organic carbon content tendency, the soil total N content increased with increasing biochar application rates in the 0–20 cm soil depth, especially in 2015. In addition to labile C and N in biochar, the increase in organic matter and total N with the biochar treatments may also be associated with the increasing yield and biomass, thereby returning more plant residues to the soil. Meanwhile, successive applications of 20 t ha^-1^ biochar increased the total N content in the 0–20 cm soil layer year by year.

The alteration of soil pH has significant implications for nutrient availability and organic soil matter mineralization, thereby affecting subsequent nutrient delivery (especially N and base cations such as Ca^2+^, Mg^2+^, and K^+^) [30]. In this study, soil pH decreased in the biochar-free treatment and increased with increasing rates of biochar addition in all three years, which was consistent with previous results [47]. This result confirmed that biochar could serve as a liming agent to improve soil pH for Inceptisols. Generally, the pH of biochar is influenced by the type of feedstock used, production temperature, and production duration [11], whereas the effectiveness of liming materials is determined by the pH buffer capacity of the soil and the neutralizing values of the amendments.

The ability of biochar to retain P in soil varied with the biochar application rate and the P concentration in the soil solution. In our study, biochar application had no significant influence on the available P content in 2013. Moreover, the soil available P content in the 0–20 cm soil layer decreased with the 20 t ha^-1^ biochar application compared with the biochar-free treatment in 2014 and 2015. Lehmann et al. [32] reported contrasting findings, observing increased available P concentrations after biochar addition in Anthrosols and Ferralsols. Parvage et al. [48] reported a similar increase in the soil available P. One possible reason for the observed decrease in the soil available P in our study may be that the large amount of free Ca^2+^, Mg^2+^, and Fe^3+^ oxides contained in the biochar served as P sorption sites [49]. Meanwhile, the P availability was highly pH-dependent, with a high solution pH helping precipitation of phosphate to less soluble forms [50]. Thus, successive biochar applications could limit P availability, but further study is required to clarify the underlying mechanism.

A high K content in the biochar contributed to more plant available K in the soil [51]. In this study, we found that biochar application improved the available K content of the 0–20 cm soil layer. It has been suggested that biochar retained K^+^ in a Typic Plinthudult soil via electrostatic attraction forces [25]. Yuan et al. [52] also reported that biochar had a greater K^+^ retaining effect, reducing K^+^ release by 7.9–23.4%.

## Conclusions

Successive applications of biochar to silt loam soil positively affected cotton growth, soil fertility, and N retention, but the effects varied with the biochar application rate and application time. Greater effects on cotton productivity and fiber quality were observed with higher rates of biochar application. The biochar amendments also significantly increased the soil organic carbon; total N, NO_3_^−^−N, and NH_4_^+^-N; and available K contents of the 0–20 cm layer. Application of biochar also decreased the contents of NO_3_^−^-N in the 60–100 cm soil profile, especially after three years of amendments. In conclusion, successive applications of biochar to soil have the potential to enhance cotton growth and arable soil fertility while reducing NO_3_^−^−N leaching in the North China Plain; however, the long-term effects of successive biochar applications on the properties of deep soil require further study.

## Supporting information

S1 Table ANOVA for the effects of year, treatment and their interaction effects soil N contents. (DOCX)

S1 Fig Schematic design of one field plot under cotton-garlic intercropping system. (DOCX)
