# Supplementary material for: Biochar derived from corn straw affected availability and distribution of soil nutrients and cotton yield
Source: PLoS One. 2018 Jan 11;13(1):e0189924. doi: 10.1371/journal.pone.0189924 (PMC5764240; doi:10.1371/journal.pone.0189924)
Supplement: S1 Fig — (DOCX) [file pone.0189924.s002.docx]

**Supplementary materials**

**Biochar derived from corn straw affected availability and distribution of soil nutrients and cotton yield**

**Xiaofei Tian^1^, Chengliang Li^1,^*****, Min Zhang^1^*****,** **YongshanWan^2^, ZhihuaXie^3^, Baocheng Chen^1^, Wenqing Li^1^**

^1^National Engineering Laboratory for Efficient Utilization of Soil and Fertilizer Resources, National Engineering, Technology Research Center for Slow and Controlled Release Fertilizers, College of Resources and Environment, Shandong Agricultural University, Tai’an, Shandong 271018, China

^2^Soil and Water Science Department, Tropical Research & Education Center, University of Florida, 18905 SW 280th Street, Homestead, FL 33031, USA

^3^Jining Academy of Agricultural Sciences, Jining, Shandong 272000, China

*Corresponding author

E-mail: chengliang_li11@163.com (C. Li); minzhang-2002@163.com (M. Zhang).

**S1 Fig** Schematic design of one field plot under cotton-garlic intercropping system

Line spacing

1.1 m

Plot width 4.4 m

0.37 m

Cotton plant

Plot length 5.0 m

Line spacing

0.2 m

Garlic plant
